# *ent*-Kaurane diterpenoids from the plant *Wedelia trilobata*

**DOI:** 10.1007/s13659-013-0029-4

**Published:** 2013-06-01

**Authors:** Bing-Ji Ma, Chun-Nan Wen, Yuan Gao, Fu-Cai Ren, Fei Wang, Ji-Kai Liu

**Affiliations:** 1 Agronomy College of Henan Agricultural University, Zhengzhou, 450002 China; 2 BioBioPha Co., Ltd., Kunming, 650201 China; 3 State Key Laboratory of Phytochemistry and Plant Resources in West China, Kunming Institute of Botany, Kunming, 650201 China

**Keywords:** *ent*-kaurane diterpenoids, *Wedelia trilobata*, phytochemical investigation

## Abstract

Four new *ent*-kaurane diterpenoids, namely, 3*α*-tigloyloxypterokaurene L_3_ (**1**), *ent*-17-hydroxy-kaura-9(11),15-dien-19-oic acid (**2**), and wedelobatins A (**3**) and B (**4**), together with 11 known *ent*-kaurane diterpenoids (**5**-**15**), were isolated from the ethanol extract of *Wedelia trilobata*. All the structures of **1–15** were elucidated on the basis of spectroscopic studies.

**Electronic Supplementary Material:**

Supplementary material is available for this article at 10.1007/s13659-013-0029-4 and is accessible for authorized users.

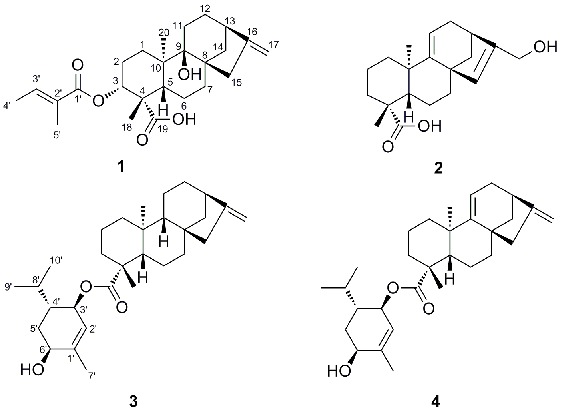

## Introduction

*Wedelia trilobata* has been used as a traditional herbal medicine for the treatment of fever and malaria in Vietnam and also for the treatment of backache, stubborn wounds, sores, and arthritic pain in the Caribbean and Central America.[1,2] Previous phytochemical studies showed that the plants of this genus are a rich source of *ent*-kaurane diterpenoids.[3–5]

As part of our efforts to assemble a large scale natural product library with thousands of compounds derived from plants and micro-organisms, phytochemical investigation on *W. trilobata* led to the isolation of four new *ent*-kaurane diterpenoids, namely, 3*α*-tigloyloxypterokaurene $L_3$ (**1**), *ent*-17-hydroxykaura-9(11),15-dien-19-oic acid (**2**), and wedelobatins A (**3**) and B (**4**), together with 11 *ent*-kaurane derivatives, grandiflorenic acid (**5**),[6] pterokaurene $L_3$ (**6**),[7] 3*α*-cinnamoyloxy-pterokaurene $L_3$ (**7**),[3] *ent*-3*β*-cinnamoyloxykaur-16-en-19-oic acid (**8**),[4] grandifloric acid (**9**),[8] *ent*-17-hydroxykaur-15-en-19-oic acid (**10**),[3] 3*α*-angeloyloxypterokaurene $L_3$ (**11**),[3] *ent*-3*β*-tigloyloxykaur-16-en-19-oic acid (**12**),[10] *ent*-3*β*-angeloyloxykaur-16-en-19-oic acid (**13**),[3,10] 12*α*-methoxygrandiflorenic acid (**14**),[11] and 12*α*-hydroxygrandiflorenic acid (**15**).[12] This paper herein describes the isolation and structural elucidation of these new compounds.

1

2

3

4

OCin =

OTig =

OAng =

| | R1 | R2 | R3 | R4 | R5 |
|---|---|---|---|---|---|
| **5** | H | $\Delta^{9(11)}$ | H | H | =$CH_2$ |
| **6** | H | OH | H | H | =$CH_2$ |
| **7** | OCin | OH | H | H | =$CH_2$ |
| **8** | OCin | H | H | H | =$CH_2$ |
| **9** | H | H | H | OH | =$CH_2$ |
| **10** | H | H | H | $\Delta^{15}$ | $CH_2OH$ |
| **11** | OAng | OH | H | H | =$CH_2$ |
| **12** | OTig | H | H | H | =$CH_2$ |
| **13** | OAng | H | H | H | =$CH_2$ |
| **14** | H | $\Delta^{9(11)}$ | $OCH_3$ | H | =$CH_2$ |
| **15** | H | $\Delta^{9(11)}$ | OH | H | =$CH_2$ |

## Results and Discussion

3*α*-Tigloyloxypterokaurene $L_3$ (**1**), was obtained as a white amorphous powder, with its molecular formula determined as $C_{25}H_{36}O_5$ on the basis of HREIMS, showing a molecular ion peak at *m/z* 416.2554 (calcd for $C_{25}H_{36}O_5$, 416.2563). The IR spectrum revealed absorption bands of hydroxyl (3513 $cm^{-1}$), carbonyl (1701 $cm^{-1}$), and double bond (1652 $cm^{-1}$) groups. In the $^1H$ NMR spectrum (Table 1), the downfield olefinic proton

*To whom correspondence should be addressed. E-mail: jkliu@mail.kib.ac.cn

**Table 1. NMR data of compounds 1 and 2 ($^{13}$C NMR, 100 MHz, $^{1}$H NMR, 600 MHz; $CDCl_3$)**

| | **1** | | **2** | |
|---|---|---|---|---|
| positon | $\delta_C$, type | $\delta_H$ (*J* in Hz) | $\delta_C$, type | $\delta_H$ (*J* in Hz) |
| 1 | 30.6, $CH_2$ | 2.03, td (13.1, 4.1); 1.63[a] | 39.8, $CH_2$ | 1.86[a]; 1.22, td (13.9, 3.3) |
| 2 | 23.8, $CH_2$ | 2.40, dddd (13.5, 13.1, 12.1, 4.1); 1.79[a] | 20.0, $CH_2$ | 1.86[a]; 1.48[a] |
| 3 | 78.7, CH | 4.60, dd (12.1, 4.5) | 38.2, $CH_2$ | 2.13, br. d (13.2); 1.01[a] |
| 4 | 48.0, C | | 44.9, C | |
| 5 | 49.2, CH | 1.79[a] | 47.2, CH | 1.48[a] |
| 6 | 21.4, $CH_2$ | 1.86, m; 1.70, m | 18.4, $CH_2$ | 2.44, br. q-*like* (11.5); 1.96, m |
| 7 | 35.9, $CH_2$ | 1.84, m; 1.28[a] | 27.1, $CH_2$ | 1.86[a]; 1.48[a] |
| 8 | 49.0, C | | 45.9, C | |
| 9 | 77.3, C | | 157.2, C | |
| 10 | 43.7, C | | 38.1, C | |
| 11 | 30.0, $CH_2$ | 1.99, br. dd (14.9. 5.6); 1.24, m | 113.5, CH | 5.00, dd (3.7, 3.1) |
| 12 | 34.4, $CH_2$ | 1.64[a]; 1.58, m | 27.9, $CH_2$ | 2.27, ddd (18.1, 5.0, 3.1); 1.92, br. dd (18.1, 3.7) |
| 13 | 42.1, CH | 2.62, br. s | 38.8, CH | 2.69, dd (5.0, 4.8) |
| 14 | 40.3, $CH_2$ | 2.13, dd (11.9, 2.4); 1.27[a] | 49.2, $CH_2$ | 1.63, br. d (9.2); 1.82, dd (9.2, 4.8) |
| 15 | 43.7, $CH_2$ | 1.80[a]; 2.70, br. d (17.8) | 137.2, CH | 6.01, s |
| 16 | 154.6, C | | 143.4, C | |
| 17 | 103.4, $CH_2$ | 4.81, br. s; 4.78, br. s | 60.7, $CH_2$ | 4.25, dd (14.1, 1.4); 4.21, dd (14.1, 0.9) |
| 18 | 24.1, $CH_3$ | 1.27, s | 28.3, $CH_3$ | 1.24, s |
| 19 | 180.2, C | | 183.6, C | |
| 20 | 17.3, $CH_3$ | 1.16, s | 23.0, $CH_3$ | 1.01, s |
| 1′ | 167.7, C | | | |
| 2′ | 128.8, C | | | |
| 3′ | 137.2, CH | 6.86, br. q (7.1) | | |
| 4′ | 14.4, $CH_3$ | 1.77, br. d (7.1) | | |
| 5′ | 12.0, $CH_3$ | 1.82, br. s | | |

[a]signal overlapped at $\delta_H$ 6.86 (br. q, *J* = 7.1 Hz) and two methyl signals at $\delta_H$ 1.77 (br. d, *J* = 7.1 Hz) and 1.82 (br. s), was indicated by the presence of a tigloyloxy group in **1**.[10] Apart from the five carbon signals assigned to the tigloyloxy group ($\delta_C$ 167.7, 128.8, 137.2, 14.4, and 12.0),[10] the $^{13}$C NMR (DEPT) spectrum (Table 1) of **1** also exhibited 20 carbons composed of two methyls, nine methylenes, three methines (one oxygenated), and six quaternary carbons, which were consistent with a skeleton of an *ent*-kauranoid.[7] In particular, the NMR spectroscopic features of **1** are similar to those of **6** (pterokaurene $L_3$), which only differed in the appearance of a tigloyloxy group at C-3 in **1**. It was also confirmed by the chemical shift value of C-3 ($\delta_C$ 78.7, CH), C-9 ($\delta_C$ 77.3, C) and the HMBC correlations (Figure 1) from H-3 ($\delta_H$ 4.60, dd, *J* = 12.1, 4.5 Hz) to C-1′ ($\delta_C$ 167.7, C), C-1 ($\delta_C$ 30.6, $CH_2$), and C-18 ($\delta_C$ 24.1, $CH_3$) as well as the correlations from Me-20, H-12, and H-15 to C-9, and from the methyl at C-4 (Me-18) to a downfield quaternary carbon (C-19) at $\delta_C$ 180.2. The $\beta$-orientation of the hydroxy group at C-9 in **1** was based on the

**Figure 1.** Key HMBC correlations of compounds **1**–**4**

downfield shift of H-15$\beta$ ($\delta_H$ 2.70) and upfield shift of C-15 ($\delta_C$ 43.7) for the $\gamma$-steric compression effect in **1** from the hydroxyl group at C-9 to H-15$\beta$ as evidenced in pterokaurene $L_3$ (**6**).[7] Furthermore, the ROESY correlations (Figure 2) of H-3 with H-5 and Me-18 suggested that the tigloyloxy was $\alpha$-orientated. Consequently, the structure of **1** was finally determined as *ent*-3$\beta$-tigloyloxy-9$\alpha$-hydroxykaur-16-en-19-oic acid, and given the name as 3$\alpha$-tigloyloxypterokaurene $L_3$.

**Figure 2.** Key ROESY correlations of compound **1**

*ent*-17-Hydroxykaura-9(11),15-dien-19-oic acid (**2**), was obtained as a white, amorphous powder, with a molecular formula of $C_{20}H_{28}O_3$ on the basis of HREIMS, showing a molecular ion peak at *m/z* 316.2028 (calcd for $C_{20}H_{28}O_3$, 316.2038). The IR spectrum indicated the presence of hydroxyl (3427 $cm^{-1}$), carbonyl (1693 $cm^{-1}$), and double bond (1639 $cm^{-1}$) groups. The $^{13}$C NMR (DEPT) spectrum (Table 1) revealed 20 carbons including three $sp^3$ quaternary carbons, three $sp^2$ quaternary carbons (one carboxylic acid carbonyl), two $sp^3$ methines, two $sp^2$ methines, eight $sp^3$ methylenes (one oxygenated), and two methyl groups. Its $^{1}$H NMR spectrum (Table 1) showed two olefinic protons at $\delta_H$ 6.01 (s) and 5.00 (dd, *J* = 3.7, 3.1 Hz), two AB double doublets assigned to the protons of a hydroxymethyl group at $\delta_H$ 4.25 (dd, *J* = 14.1, 1.4 Hz) and 4.21 (dd, *J* = 14.1, 0.9 Hz), and two methyl signals at $\delta_H$ 1.24 and 1.01 (each 3H, s). These spectroscopic features suggested that the structure of **2** was similar to that of **10** (*ent*-17-hydroxykaur-15-en-19-oic acid),[3] and only differed in appearance as a double bond between C-9 ($\delta_C$ 157.2, C) and C-11 ($\delta_C$ 113.5, CH) in **2**. It was confirmed by, the HMBC correlations from H-11 ($\delta_H$ 5.00, dd, $J$ = 3.7, 3.1 Hz) to C-8 ($\delta_C$ 45.9, C), C-10 ($\delta_C$ 38.1, C), and C-13 ($\delta_C$ 38.8, CH), as shown in Figure 1. The $\alpha$-orientation of the carboxylic acid group at C-4 was inferred from the $^{13}$C NMR chemical shift of the methyl group at C-4 by comparing those of related *ent*-kaurane diterpenoids, in which the methyl group with $\beta$-orientation resulted in resonance of approximately $\delta_C$ 29, as opposed to resonance of approximately $\delta_C$ 16 when the methyl group was in the $\alpha$-orientation.[7,13,14] Accordingly, the structure of compound **2** was elucidated as *ent*-17-hydroxykaura-9(11),15-dien-19-oic acid.

Wedelobatin A (**3**), was obtained as a colorless oil, which has a molecular formula of $C_{30}H_{46}O_3$ on the basis of HREIMS, showing a molecular ion peak at *m/z* 454.3454 (calcd for $C_{30}H_{46}O_3$, 454.3447). The IR spectrum suggested the presence of hydroxyl (3428 cm$^{-1}$), carbonyl (1719 cm$^{-1}$), and double bond (1657 cm$^{-1}$) groups. In the $^{13}$C NMR (DEPT) spectrum (Table 2), 20 carbon signals including two methyl carbons, nine methylenes, three methines, four quaternary carbons, and two carbons of one double bond, suggested the presence of an *ent*-kaurene skeleton, which was confirmed by the typical $^1$H NMR signals (Table 2) of *ent*-kaurene as follows: $\delta$ 4.79 (1H, br. s), 4.73 (1H, br. s), 2.63 (1H, br. s), 1.18 (3H, s), and 0.91 (3H, s). Particularly, the NMR signals of the *ent*-kaurane moiety were in accordance with those of *ent*-kaurenoic acid.[7] On the other hand, the remaining carbon signals were composed of two olefinic carbons of a trisubstituted double bond, three methyl carbons, one methylene, and four methines (two oxygenated), together with the $^1$H NMR signals at $\delta$ 5.43 (1H, br. s), 5.15 (1H, br. d, $J$ = 8.4 Hz), 4.02 (1H, t, $J$ = 3.3 Hz), 1.81 (3H, s), 0.96, and 0.83 (each 3H, d, $J$ = 6.8 Hz), resembled those of (3*R*,4*R*,6*S*)-3,6-dihydroxymenth-1-ene.[15,16] As shown in Figure 3, the cyclohexene ring in the monoterpene moiety should have half chair configuration. The coupling constant of H-3′ (br. d, $J$ = 8.4 Hz) indicated a *trans* pseudo-diaxial relationship for H-3′ and H-4′ while those of H-6′ (t, $J$ = 3.3 Hz) suggested an equatorial orientation. Consequently, H-3′, H-4′, and H-6′ were determined to be $\alpha$-, $\beta$-, and $\alpha$-oriented, respectively. The observation of the HMBC correlation (Figure 1) from H-3′ to C-19 as well as the down-field chemical shift of H-3′ at $\delta_H$ 5.15 and the upfield chemical shift of C-19 at $\delta_C$ 177.4, indicated the linkage between the two moieties at C-3′ and C-19 via an ester connection. Therefore, the structure of wedelobatin A was elucidated, as shown in Figure 1.

**Figure 3.** Configuration of the monoterpene moiety

Wedelobatin B (**4**), was obtained as a colorless oil, and determined molecular formula of $C_{30}H_{44}O_3$ on the basis of HREIMS, showing a molecular ion peak at *m/z* 452.3283 (calcd for $C_{30}H_{44}O_3$, 452.3290). The IR spectrum suggested the presence of hydroxyl (3429 cm$^{-1}$), carbonyl (1716 cm$^{-1}$), and double bond (1654 cm$^{-1}$) groups. The $^1$H and $^{13}$C NMR data (Table 2) of **4** were similar to those of **3**. The major difference found was the presence of one more olefinic proton at $\delta_H$ 5.23 (dd, $J$ = 3.5, 2.7 Hz) and two olefinic carbons of a trisubstituted double bond in **4**. The double bond was located at C-9 based on the HMBC correlations from H-11 to C-8, C-10, and C-13, which was confirmed by the fact that the NMR data of the *ent*-kaurene moiety were consistent with those of **5**. Hence, the structure of wedelobatin B was determined, as shown in Figure 1.

## Experimental Section

**General Experimental Procedures.** Optical rotations were measured on a Jasco P-1020 polarimeter. IR spectra were obtained by using a Bruker Tensor 27 FT-IR spectrometer with KBr pellets. NMR spectra were acquired with a Bruker AVANCE III-600, Bruker DRX-500 or Bruker AV-400 instrument at room temperature. ESIMS and HREIMS were recorded on a Bruker HCT/Esquire and Waters AutoSpec-P776 mass spectrometers. Silica gel (200–300 mesh, Qingdao Marine Chemical Co., Ltd., China), MCI gel CHP-20P (75–150 $\mu$m, Mitsubishi Chemical Corporation, Japan), Sephadex LH-20 (Amersham Biosciences, Sweden) and Chromatorex C-18 (40–75 $\mu$m, Fuji Silysia Chemical Ltd., Japan) were used for normal pressure column chromatography. MPLC was performed on a Büchi Sepacore System including pump manager C-615, pump modules C-605, and fraction collector C-660 (Büchi Labortechnik AG, Switzerland) and columns were packed with Chromatorex C-18 (40–75 $\mu$m, Fuji Silysia Chemical Ltd., Japan). Preparative HPLC was performed on an Agilent 1200 liquid chromatography apparatus with a Zorbax SB-C18 column (5 $\mu$m, 9.4 mm × 150 mm). Fractions were monitored and analyzed by TLC (Qingdao Marine Chemical Co., Ltd., China) and spots were visualized by heating silica gel plates immersed in vanillin–$H_2SO_4$ in EtOH.

**Plant Material.** The whole plants of *W. trilobata* were collected in Pu'er City of Yunnan Province, China, in November 2010, and identified by Mr. Yu Chen of Kunming Institute of Botany, Chinese Academy of Sciences. The voucher specimen (BBP0311) was deposited at BioBioPha Co., Ltd.

**Extraction and Isolation.** The air-dried, powdered whole plants of *W. trilobata* (10.0 kg) were extracted with 95% ethanol at room temperature. The alcohol extract was concentrated to derive a residue (1180 g), which was fractionalized by silica gel column chromatography eluted with a solvent system of petroleum ether (PE)-acetone and then MeOH to yield fractions 1–6. Fraction 1 (30 g), eluted with 10% acetone, was further isolated and purified by recrystallization from PE-acetone to afford **5** (17.5 mg). Fraction 2 (48 g), eluted with 15% acetone, was further separated by silica gel column ($CHCl_3$-acetone, 60:1), and then by preparative HPLC ($CH_3CN$-$H_2O$, 50%→70%, 10 mL/min) to derive **3** (4 mg), **4** (4 mg), **12** (48 mg), and **13** (8 mg). Fraction 3 (28 g), was eluted using 20% acetone, and

**Table 2. NMR data of compounds 3 and 4 ($^{13}$C NMR, 100 MHz, $^{1}$H NMR, 500 MHz; $CDCl_3$)**

| | 3 | | 4 | |
|---|---|---|---|---|
| positon | $\delta_C$, type | $\delta_H$ (*J* in Hz) | $\delta_C$, type | $\delta_H$ (*J* in Hz) |
| 1 | 40.7, $CH_2$ | 1.84[a]<br>0.79[a] | 40.8, $CH_2$ | 1.92[a]<br>1.24[a] |
| 2 | 19.4, $CH_2$ | 1.81[a]; 1.41[a] | 20.4, $CH_2$ | 1.80[a]; 1.48[a] |
| 3 | 38.0, $CH_2$ | 2.17, br. d (13.6); 0.99[a] | 38.6, $CH_2$ | 2.15, br. d (13.4); 0.99[a] |
| 4 | 44.1, C | | 45.1, C | |
| 5 | 57.2, CH | 1.02[a] | 46.8, CH | 1.65[a] |
| 6 | 21.9, $CH_2$ | 1.84[a]; 1.80[a] | 18.6, $CH_2$ | 2.52, br. q-*like* (11.5); 1.85[a] |
| 7 | 41.3, $CH_2$ | 1.50, m; 1.43[a] | 29.7, $CH_2$ | 1.97[a]; 1.46[a] |
| 8 | 44.2, C | | 42.3, C | |
| 9 | 54.9, CH | 1.06[a] | 156.1, C | |
| 10 | 39.58, C | | 38.8, C | |
| 11 | 18.4, $CH_2$ | 1.52–1.60 (2H, m) | 114.8, CH | 5.23, dd (3.5, 2.7) |
| 12 | 33.1, $CH_2$ | 1.60[a]; 1.46, m | 38.0, $CH_2$ | 2.42, ddd (17.0, 4.4, 2.7); 1.98, m |
| 13 | 43.8, CH | 2.63, br. s | 41.3, CH | 2.77, br. s |
| 14 | 39.57, $CH_2$ | 1.95, dd (11.4, 1.3); 1.13, dd (11.4, 4.9) | 45.0, $CH_2$ | 1.60[a]; 1.51, m |
| 15 | 49.0, $CH_2$ | 2.05 (2H, m) | 50.3, $CH_2$ | 2.61, br. d (15.6); 2.18, dt (15.6, 2.4) |
| 16 | 155.9, C | | 158.6, C | |
| 17 | 102.9, $CH_2$ | 4.79, br. s; 4.73, br. s | 105.4, $CH_2$ | 4.91, 4.79, br. s |
| 18 | 29.4, $CH_3$ | 1.18, s | 28.6, $CH_3$ | 1.19, s |
| 19 | 177.4, C | | 177.2, C | |
| 20 | 16.2, $CH_3$ | 0.91, s | 24.2, $CH_3$ | 0.99, s |
| 1′ | 138.7, C | | 138.7, C | |
| 2′ | 124.9, CH | 5.43, br. s | 125.1, CH | 5.43, br. s |
| 3′ | 71.3, CH | 5.15, br. d (8.4) | 71.4, CH | 5.17, br. d (8.4) |
| 4′ | 39.2, CH | 1.83[a] | 39.2, CH | 1.84[a] |
| 5′ | 29.8, $CH_2$ | 1.83[a]; 1.60[a] | 30.0, $CH_2$ | 1.84[a]; 1.59[a] |
| 6′ | 67.7, CH | 4.02, t (3.3) | 67.7, CH | 4.02, t (3.5) |
| 7′ | 20.4, $CH_3$ | 1.81, s | 20.3, $CH_3$ | 1.80, s |
| 8′ | 26.4, CH | 1.87[a] | 26.4, CH | 1.85[a] |
| 9′ | 17.1, $CH_3$ | 0.83, d (6.8) | 17.1, $CH_3$ | 0.81, d (6.7) |
| 10′ | 20.9, $CH_3$ | 0.96, d (6.8) | 20.9, $CH_3$ | 0.96, d (6.7) |

[a]signal overlapped subsequently subjected to a silica gel column with a gradient elution (PE-acetone, 40:1→15:1) to yield fractions 3a–3c. Fraction 3a (6.5 g) yielded **6** (40 mg) and **8** (166 mg) after passing over a MCI gel (MeOH, 100%) and MPLC (MeOH-$H_2O$, 75%→82%, 10 mL/min). **1** (24 mg) and **11** (70 mg) were purified from fraction 3b (7.8 g) through a silica gel column (PE-acetone, 8:1). Fraction 3c (8.5 g) was further separated by silica gel (PE-acetone, 8:1), RP-18 (MeOH-$H_2O$, 70%), and silica gel-$AgNO_3$ (PE-acetone, 3:1→2:1) to yield **2** (16 mg), **7** (170 mg), **9** (25 mg), and **10** (9 mg). Fraction 5 (4.2 g), was eluted using 30% acetone, and purified using a silica gel column (PE-EtOAc, 4:1→3:1) to afford **14** (8 mg) and **15** (22 mg).

**3*α*-Tigloyloxypterokaurene $L_3$ (1):** amorphous powder; $[\alpha]_D^{19}$ – 72.9 (*c* 0.24, $CHCl_3$); IR (KBr) $\nu_{max}$ 3513, 2943, 2933, 2859, 1701, 1652, 1450, 1380, 1274, 1258, 1209, 1170, 1152, 1133, 1041, 970 $cm^{-1}$; $^{1}$H NMR and $^{13}$C NMR data, see Table 1; ESIMS (pos.) *m/z* 439 $[M + Na]^+$; HREIMS *m/z* 416.2554 (calcd for $C_{25}H_{36}O_5$, 416.2563).

***ent*-17-Hydroxykaura-9(11),15-dien-19-oic acid (2):** amorphous powder; $[\alpha]_D^{19}$ – 43.6 (*c* 0.25, $CHCl_3$); IR (KBr) $\nu_{max}$ 3427, 3031, 2928, 2969, 1693, 1639, 1464, 1377, 1228, 1158, 1118, 1003, 988 $cm^{-1}$; $^{1}$H NMR and $^{13}$C NMR data, see Table 1; ESIMS (neg.) *m/z* 315 $[M - H]^-$, HREIMS *m/z* 316.2028 (calcd for $C_{20}H_{28}O_3$, 316.2038).

**Wedelobatin A (3):** oil; $[\alpha]_D^{22}$ – 121.8 (*c* 0.41, $CHCl_3$); IR (KBr) $\nu_{max}$ 3428, 2956, 2929, 2872, 2853, 1719, 1657, 1465, 1447, 1386, 1369, 1227, 1149, 1015 $cm^{-1}$; $^{1}$H NMR and $^{13}$C NMR data, see Table 2; ESIMS (pos.) *m/z* 477 $[M + Na]^+$, HREIMS *m/z* 454.3454 (calcd for $C_{30}H_{46}O_3$, 454.3447).

**Wedelobatin B (4):** oil; $[\alpha]_D^{21}$ – 29.2 (*c* 0.31, $CHCl_3$); IR (KBr) $\nu_{max}$ 3429, 2957, 2929, 2870, 1716, 1654, 1464, 1377, 1219, 1144, 1045, 1015, 985 $cm^{-1}$; $^{1}$H NMR and $^{13}$C NMR data, see Table 2; ESIMS (pos.) *m/z* 475 $[M + Na]^+$, HREIMS *m/z* 452.3283 (calcd for $C_{30}H_{44}O_3$, 452.3290).

## Electronic Supplementary Material

Supplementary material is available in the online version of this article at http://dx.doi.org/10.1007/s13659-013-0029-4 and is accessible for authorized users.

## Acknowledgments

The authors acknowledge the National Basic Research Program of China (973 Program, 2009CB522300), and the "West Light" program of Chinese Academy of Sciences.

**Open Access** This article is distributed under the terms of the Creative Commons Attribution License which permits any use, distribution, and reproduction in any medium, provided the original author(s) and source are credited.

## Electronic supplementary material

Supplementary material, approximately 2.16 MB.
