# Supplementary material for: ent-Kaurane diterpenoids from the plant Wedelia trilobata
Source: Nat Prod Bioprospect. 2013 Jun 1;3(3):107–11. doi: 10.1007/s13659-013-0029-4 (PMC4131666; doi:10.1007/s13659-013-0029-4)

Electronic Supplementary Material

ent-Kaurane diterpenoids from the plant *Wedelia trilobata*

Bing-Ji Ma,[a] Chun-Nan Wen,[a,b] Yuan Gao,[b,c] Fu-Cai Ren,[b] Fei Wang,[b,c] and Ji-Kai Liu[c,]*

[a]Agronomy College of Henan Agricultural University, Zhengzhou 450002, China

[b]BioBioPha Co., Ltd., Kunming 650201, China

[c]State Key Laboratory of Phytochemistry and Plant Resources in West China, Kunming Institute of Botany, Chinese Academy of Sciences, Kunming 650201, China

Received 28 March 2013; Accepted 13 May 2013

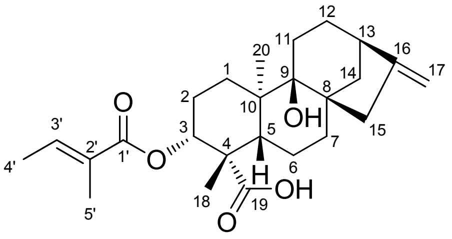

1

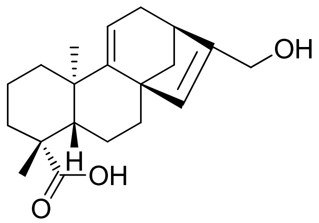

2

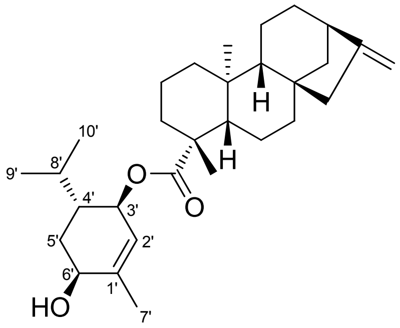

3

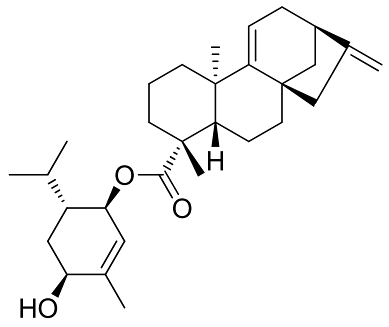

4

Structures of compounds **1**–**4**

*To whom correspondence should be addressed. E-mail: jkliu@mail.kib.ac.cn

Content list

S1. ^{1}H NMR (600 MHz) for 3*α*-tigloyloxypterokaurene L_3 (**1**)

S2. ^{13}C NMR (DEPT 100 MHz) for 3*α*-tigloyloxypterokaurene L_3 (**1**)

S3. HSQC (600 MHz) for 3*α*-tigloyloxypterokaurene L_3 (**1**)

S4. HMBC (600 MHz) for 3*α*-tigloyloxypterokaurene L_3 (**1**)

S5. ROESY (600 MHz) for 3*α*-tigloyloxypterokaurene L_3 (**1**)

S6. ^{1}H NMR (600 MHz) for *ent*-17-hydroxykaura-9(11),15-dien-19-oic acid (**2**)

S7. ^{13}C NMR (DEPT 100 MHz) for ent-17-hydroxykaura-9(11),15-dien-19-oic acid (**2**)

S8. HSQC (600 MHz) for *ent*-17-hydroxykaura-9(11),15-dien-19-oic acid (**2**)

S9. HMBC (600 MHz) for *ent*-17-hydroxykaura-9(11),15-dien-19-oic acid (**2**)

S10. ^{1}H NMR (500 MHz) for wedelobatin A (**3**)

S11. ^{13}C NMR (DEPT 100 MHz) for wedelobatin A (**3**)

S12. HSQC (600 MHz) for wedelobatin A (**3**)

S13. HMBC (500 MHz) for wedelobatin A (**3**)

S14. ^{1}H NMR (500 MHz) for wedelobatin B (**4**)

S15. ^{13}C NMR (DEPT 125 MHz) for wedelobatin B (**4**)

S16. HSQC (600 MHz) for wedelobatin B (**4**)

S17. HMBC (500 MHz) for wedelobatin B (**4**)

S1. ^{1}H NMR (600 MHz) for 3α-tigloyloxypterokaurene L$_3$ (**1**)

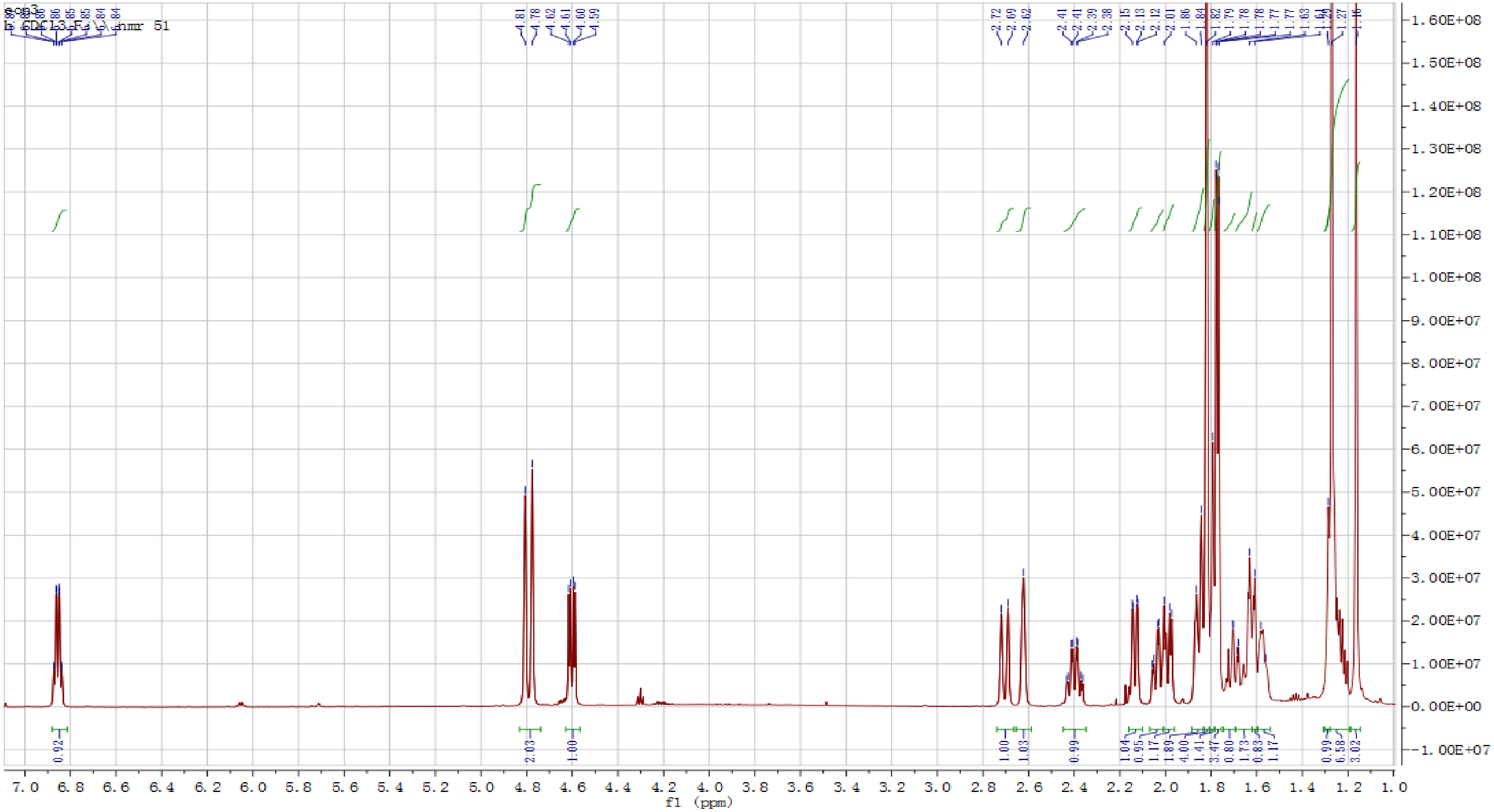

S2. ^{13}C NMR (DEPT 100 MHz) for 3α-tigloyloxypterokaurene L_3 (**1**)

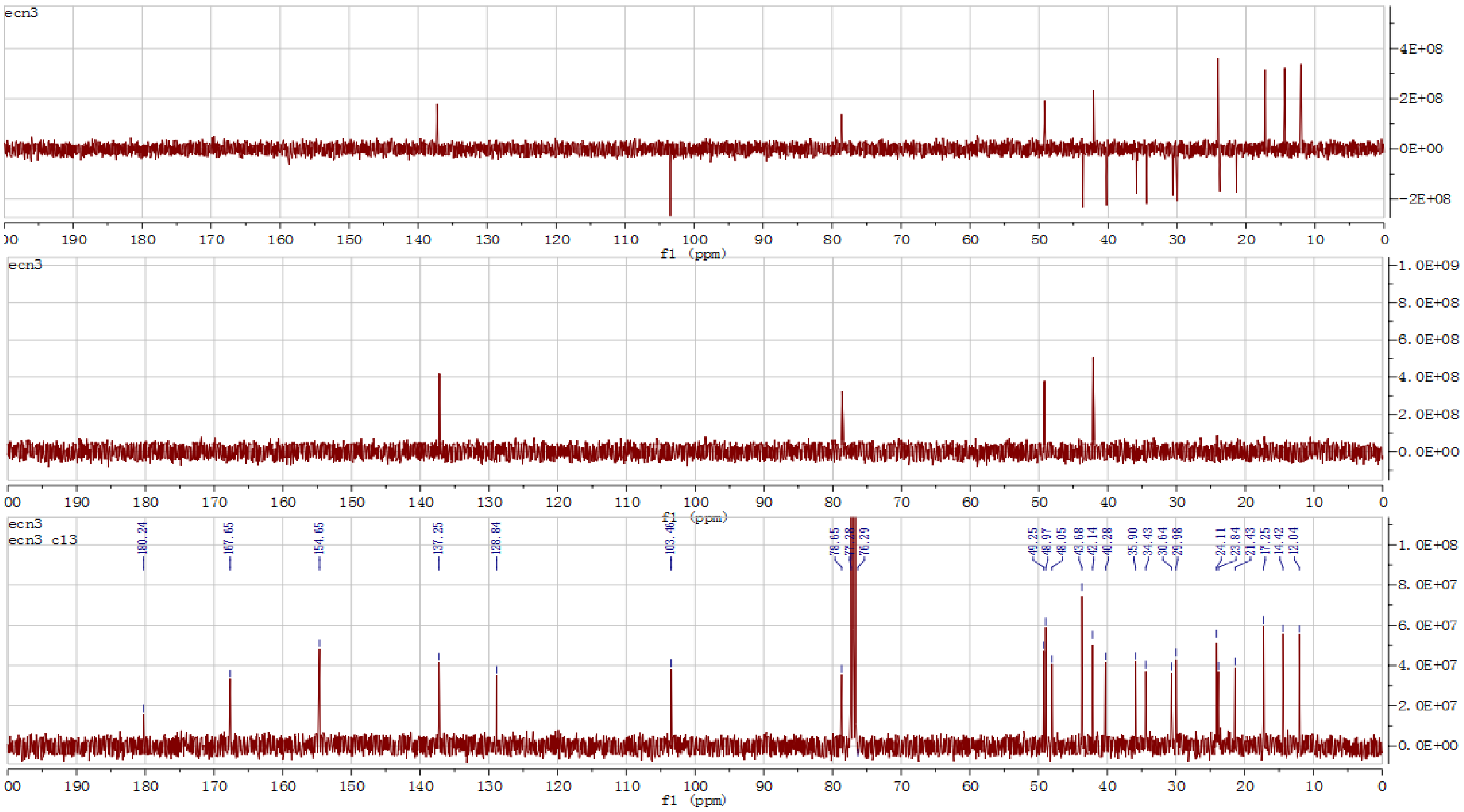

S3. HSQC (600 MHz) for 3α-tigloyloxypterokaurene L_3 (**1**)

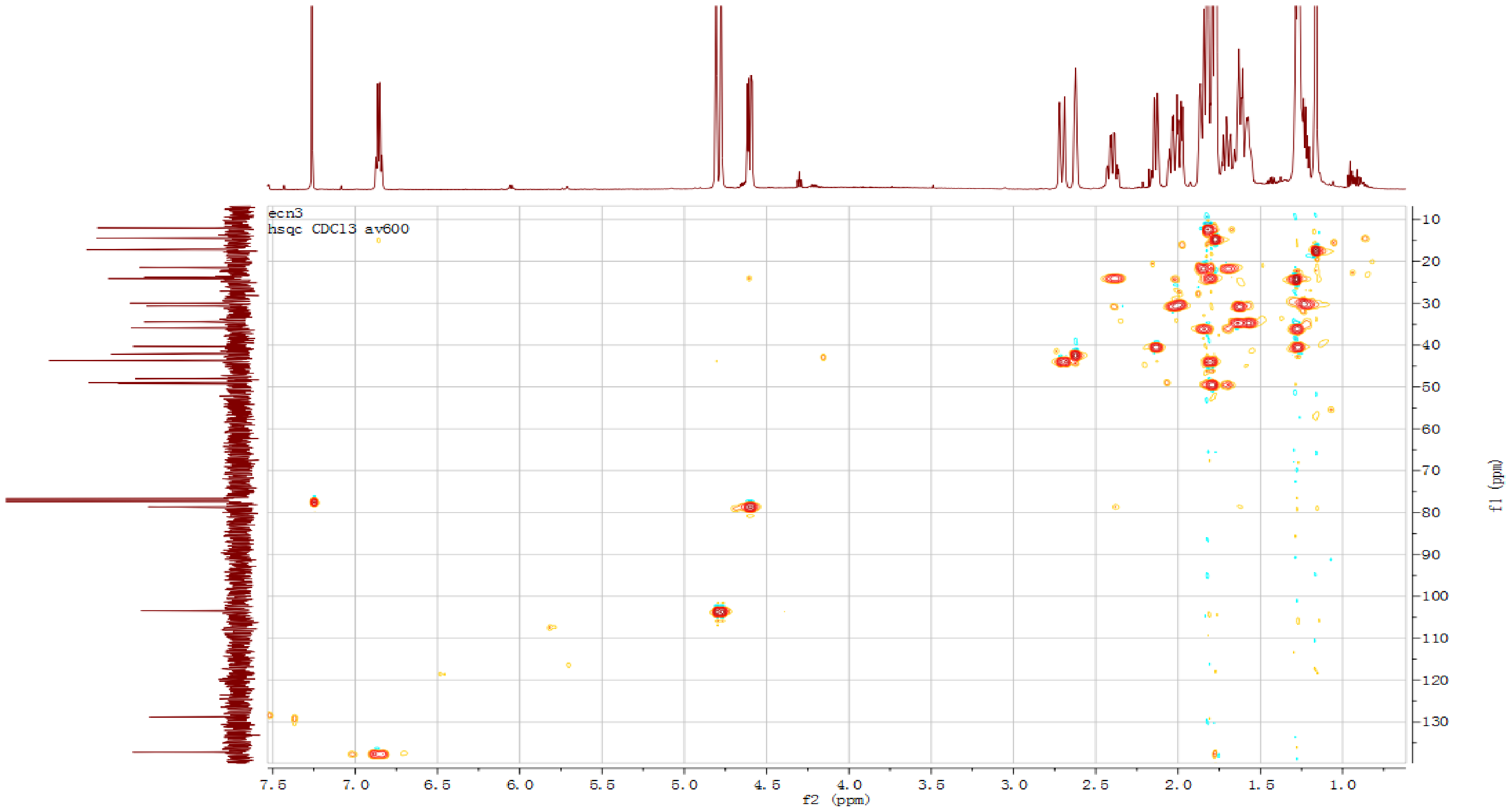

S4. HMBC (600 MHz) for 3α-tigloyloxypterokaurene L_3 (**1**)

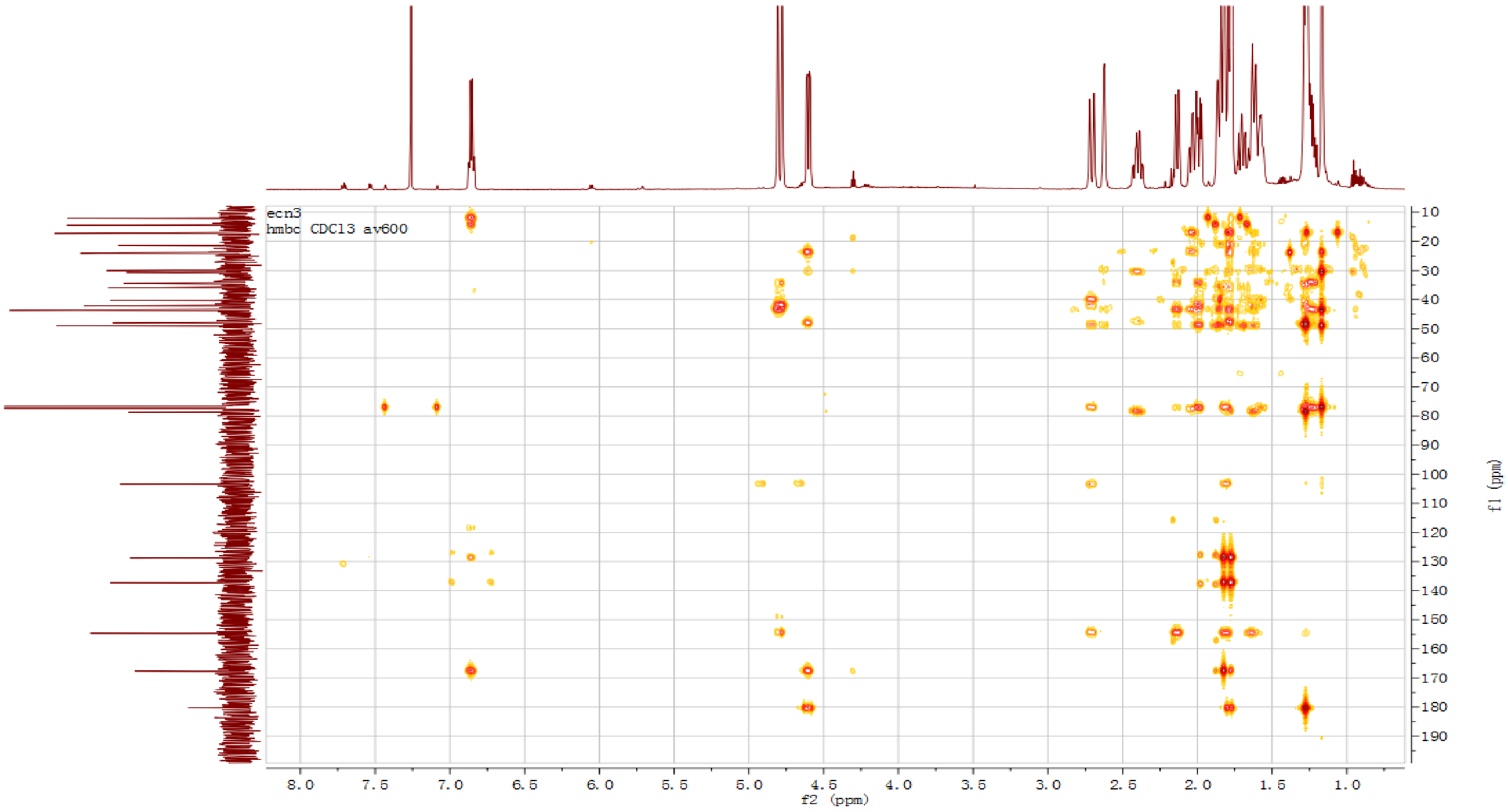

S5. ROESY (600 MHz) for 3α-tigloyloxypterokaurene L_3 (**1**)

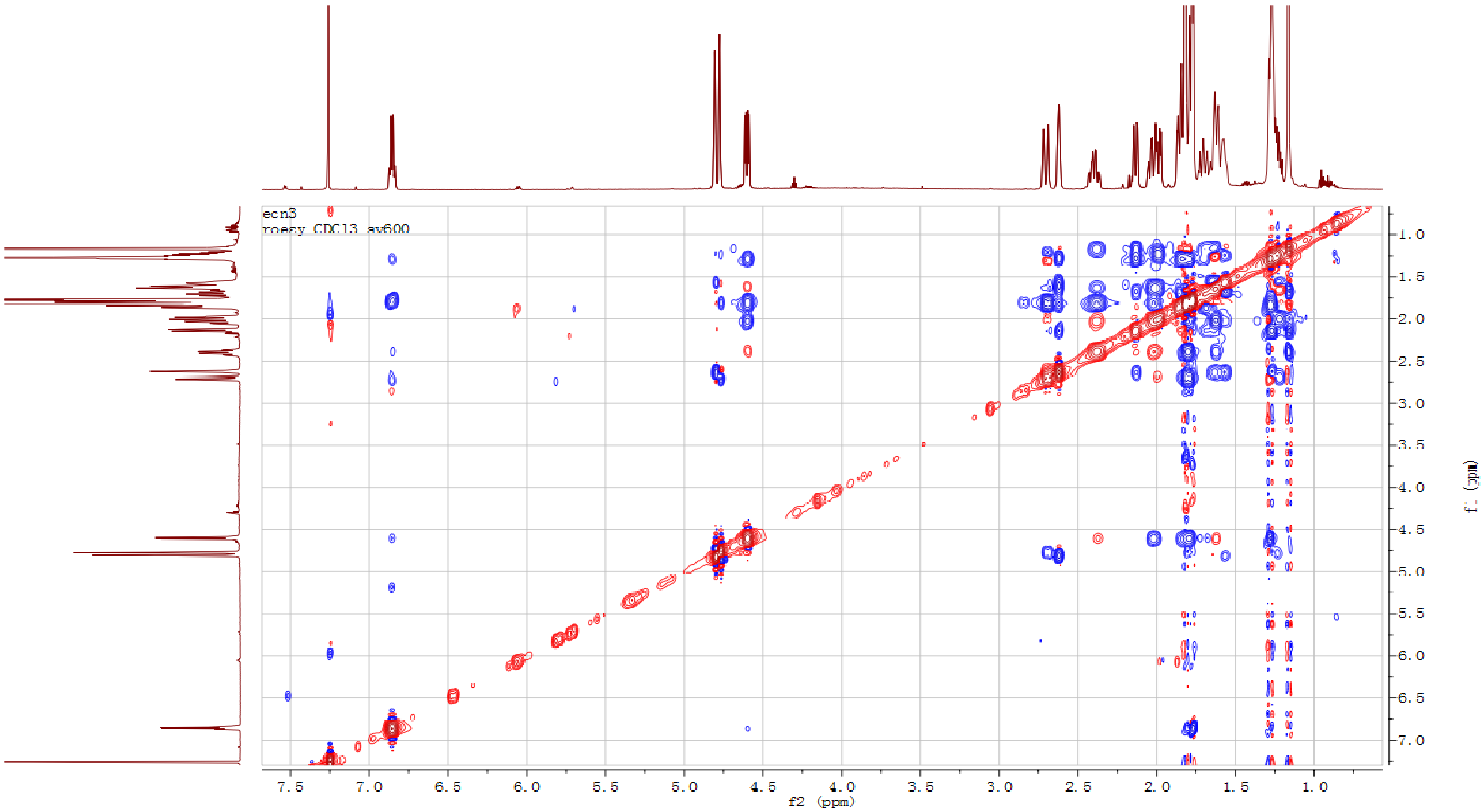

S6. ^{1}H NMR (600 MHz) for *ent*-17-hydroxykaura-9(11),15-dien-19-oic acid (**2**)

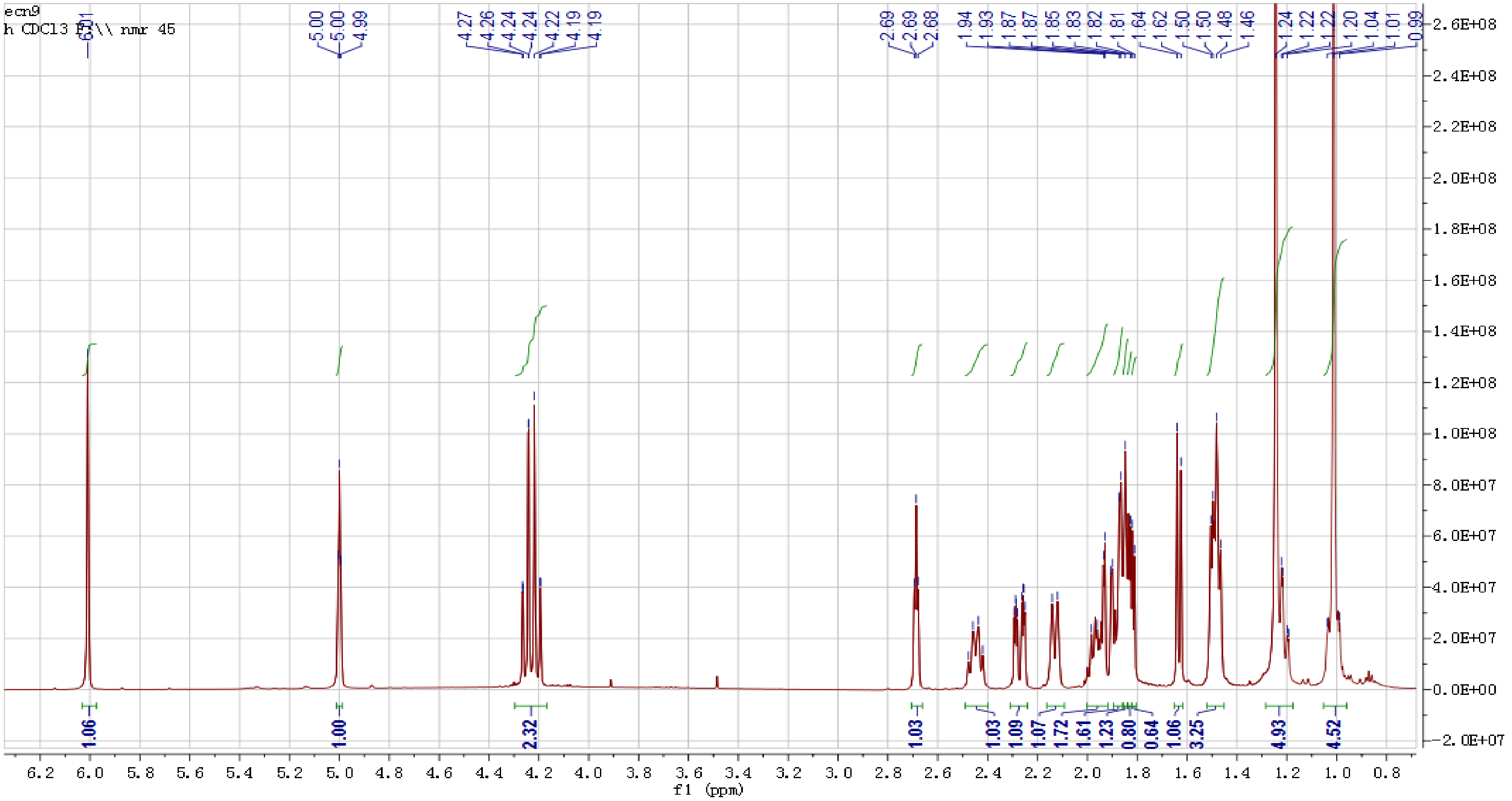

S7. ^{13}C NMR (DEPT 100 MHz) for ent-17-hydroxykaura-9(11),15-dien-19-oic acid (**2**)

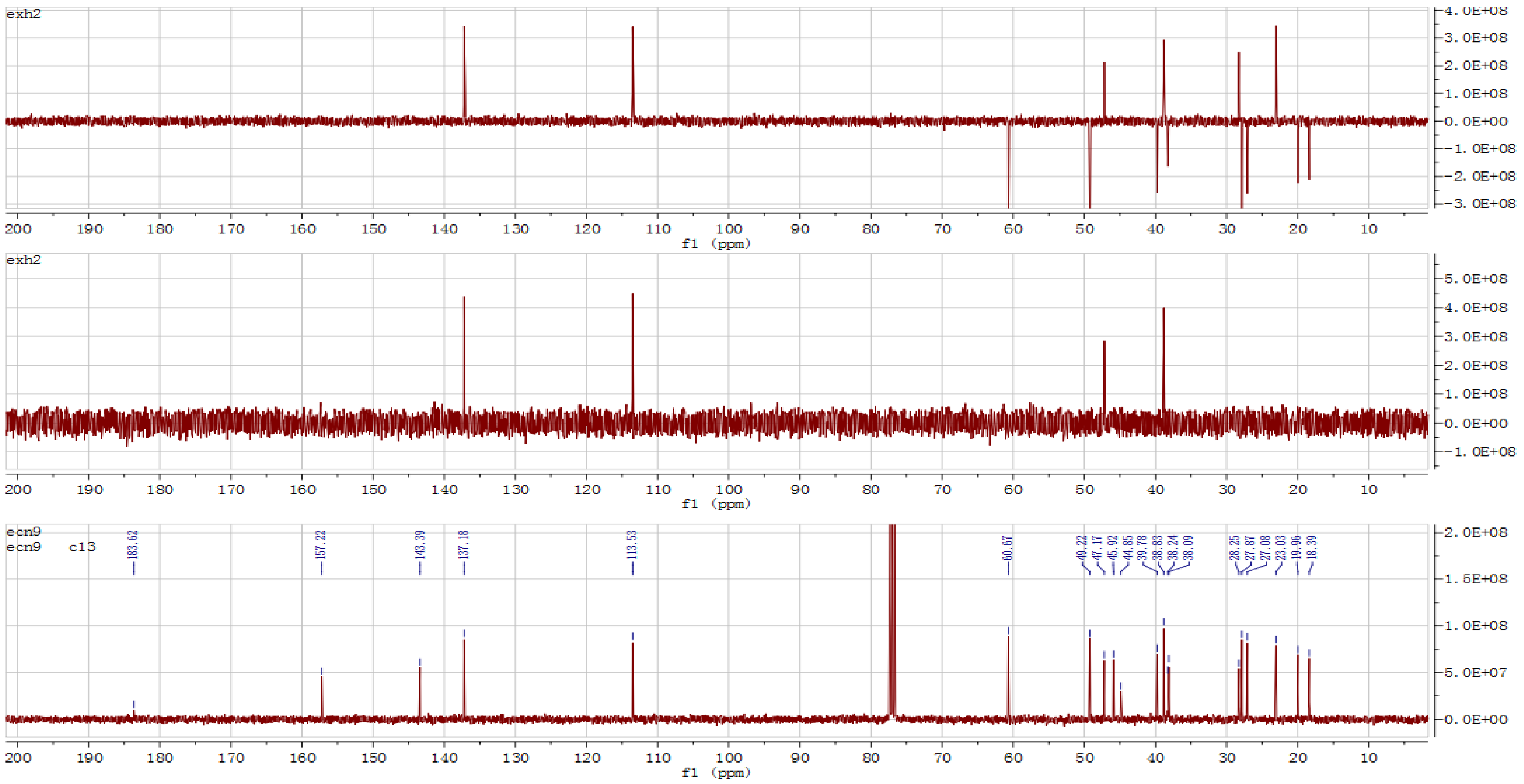

S8. HSQC (600 MHz) for *ent*-17-hydroxykaura-9(11),15-dien-19-oic acid (**2**)

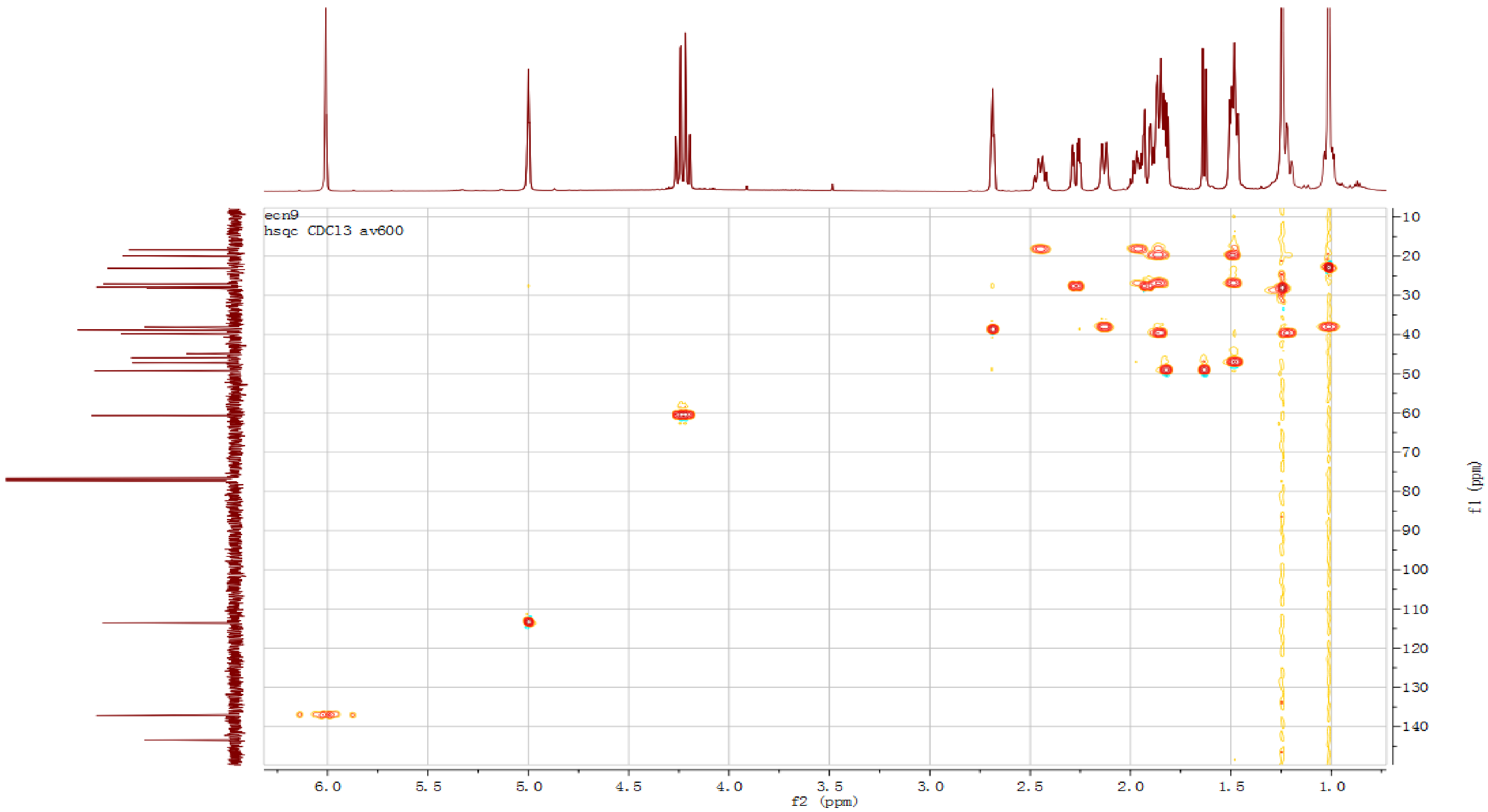

S9. HMBC (600 MHz) for *ent*-17-hydroxykaura-9(11),15-dien-19-oic acid (**2**)

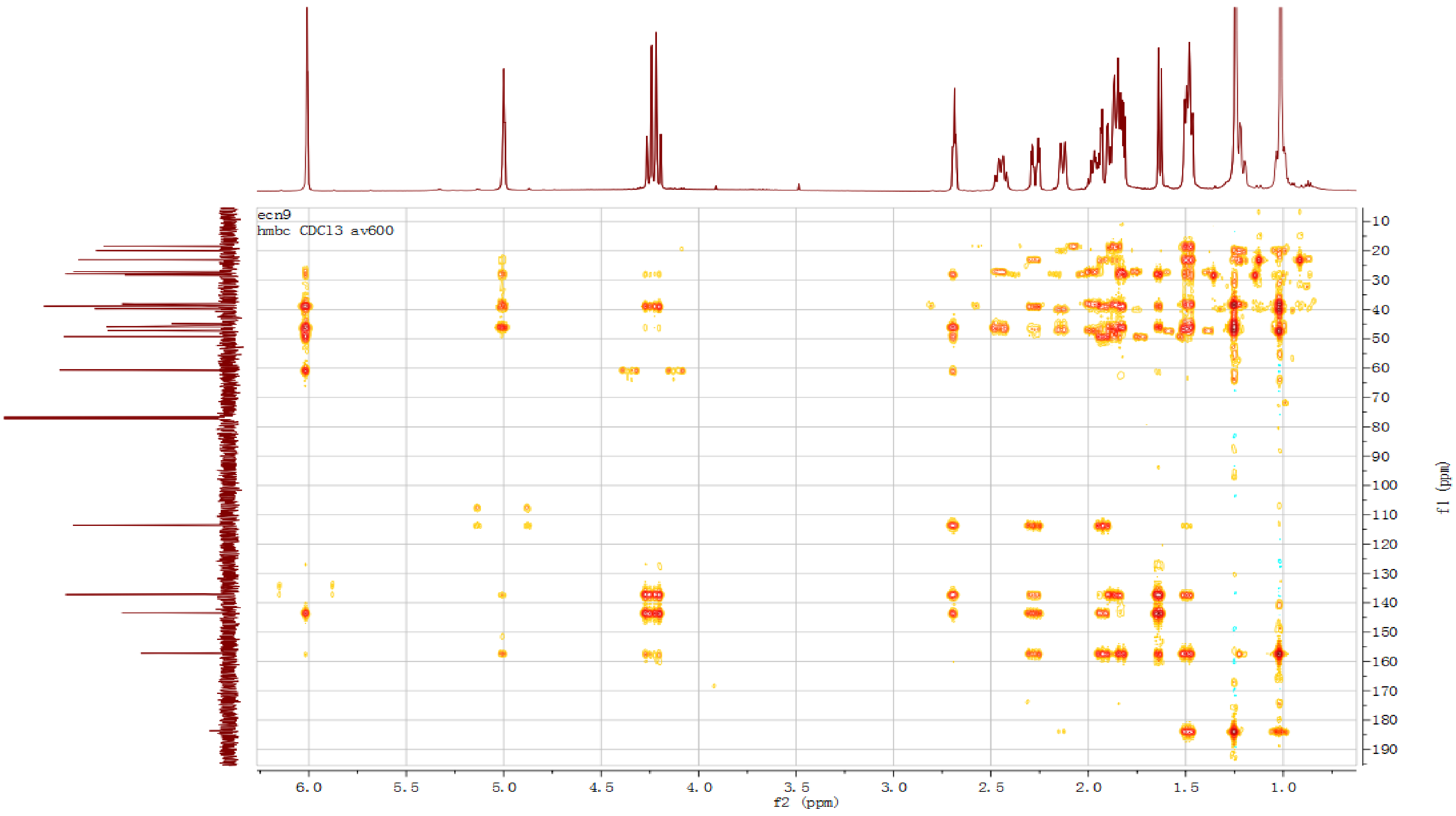

S10. ^{1}H NMR (500 MHz) for wedelobatin A (**3**)

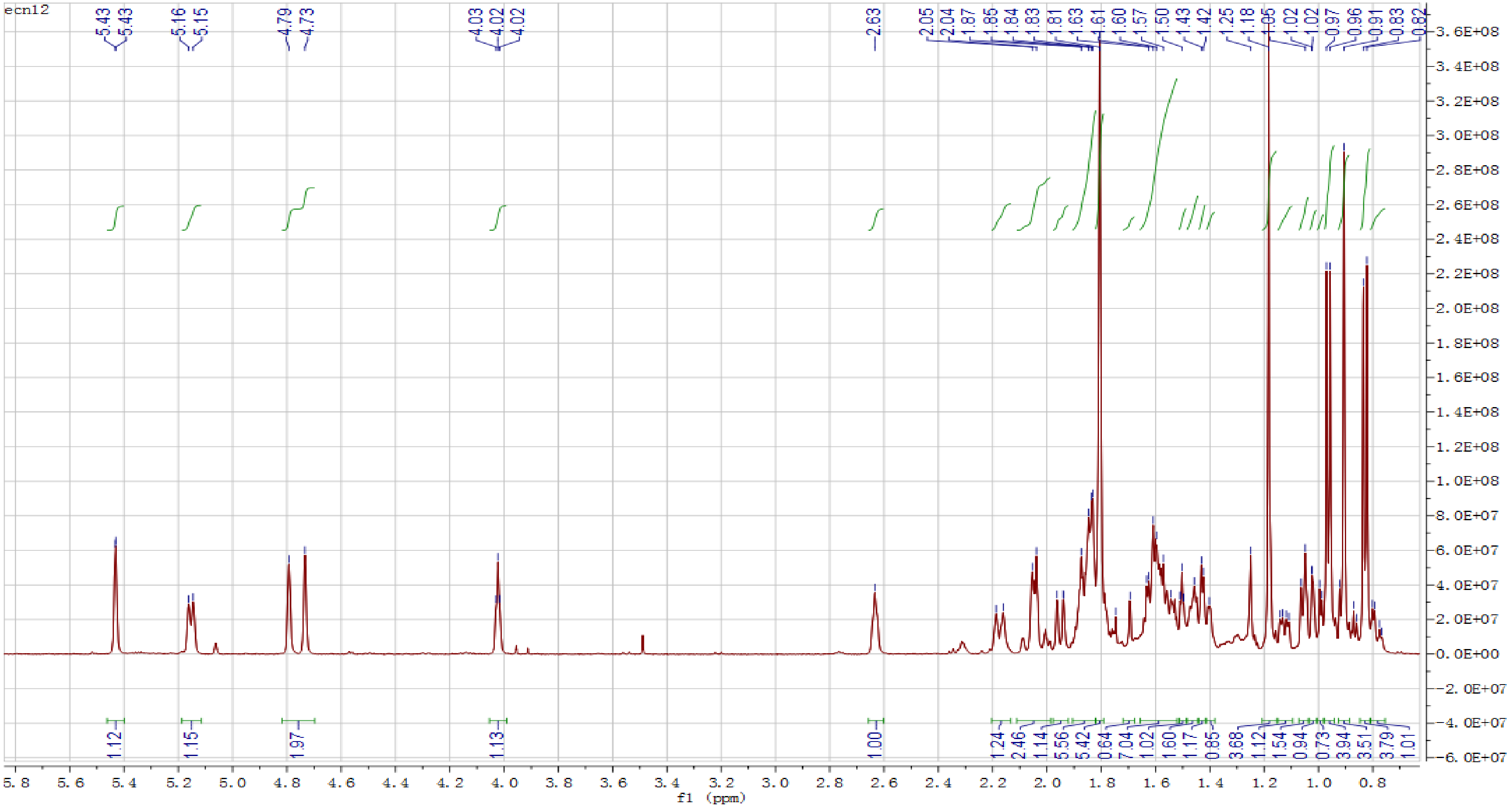

S11. ^{13}C NMR (DEPT 100 MHz) for wedelobatin A (**3**)

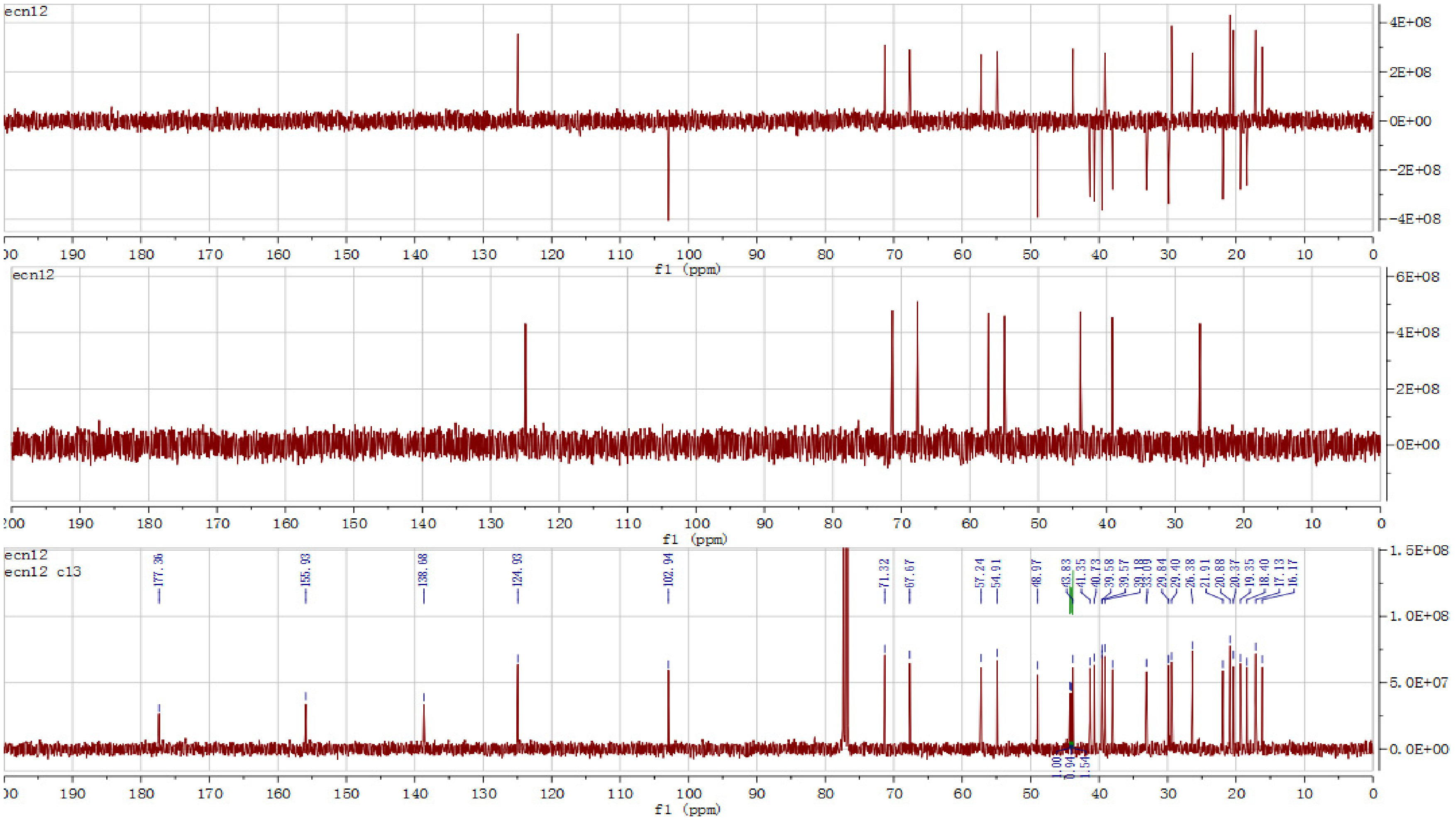

S12. HSQC (600 MHz) for wedelobatin A (**3**)

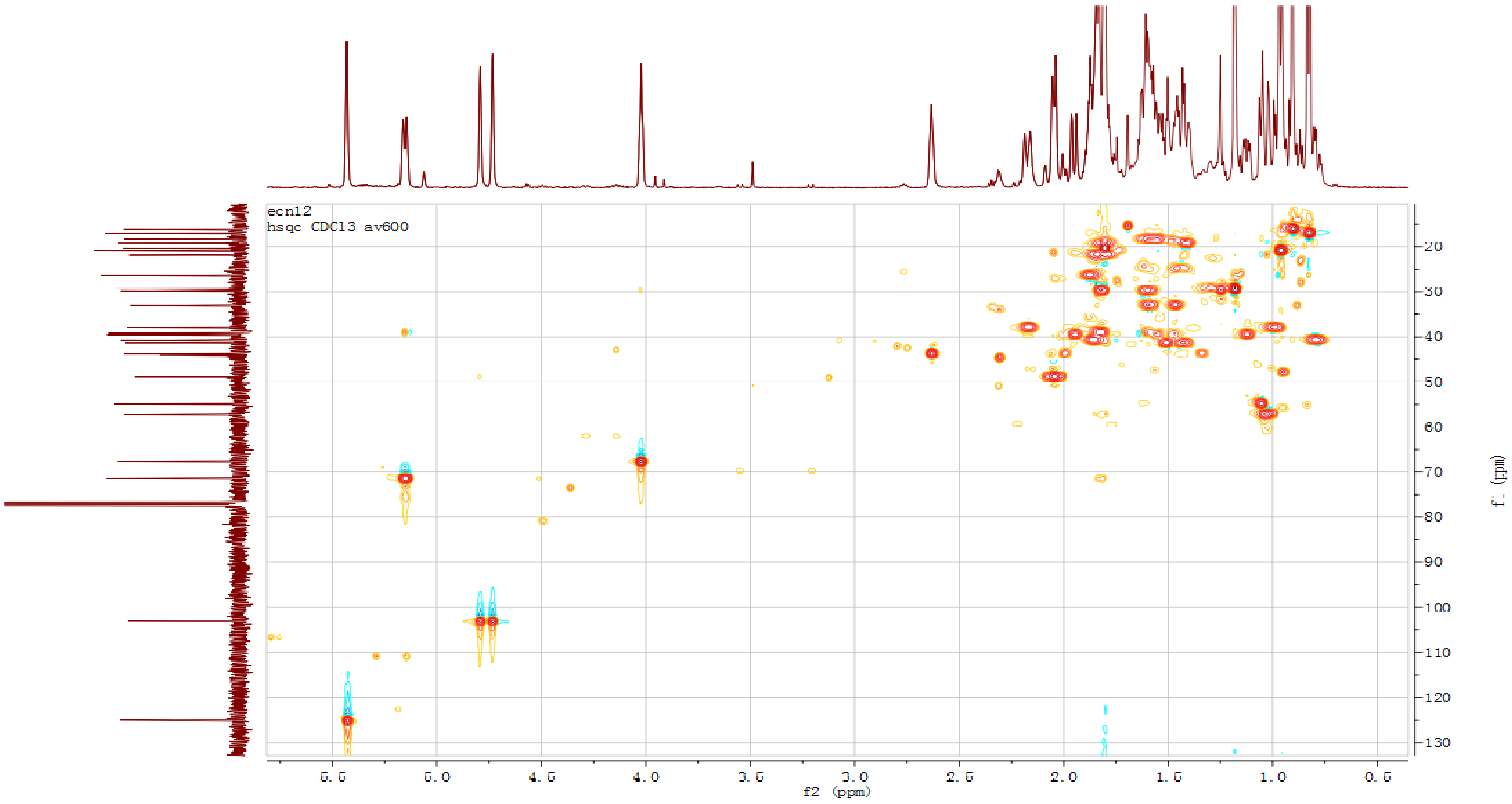

S13. HMBC (500 MHz) for wedelobatin A (**3**)

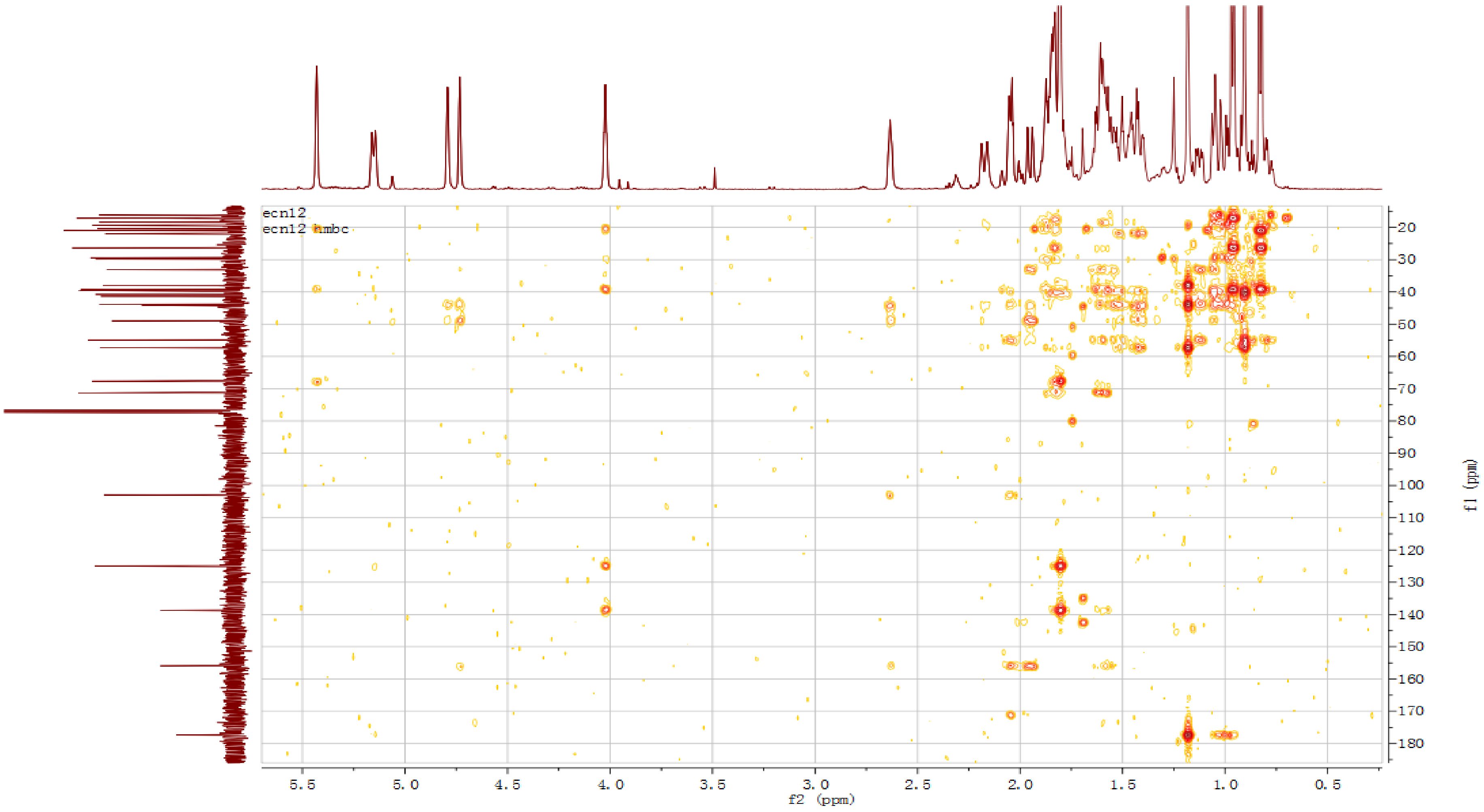

S14. ^{1}H NMR (500 MHz) for wedelobatin B (**4**)

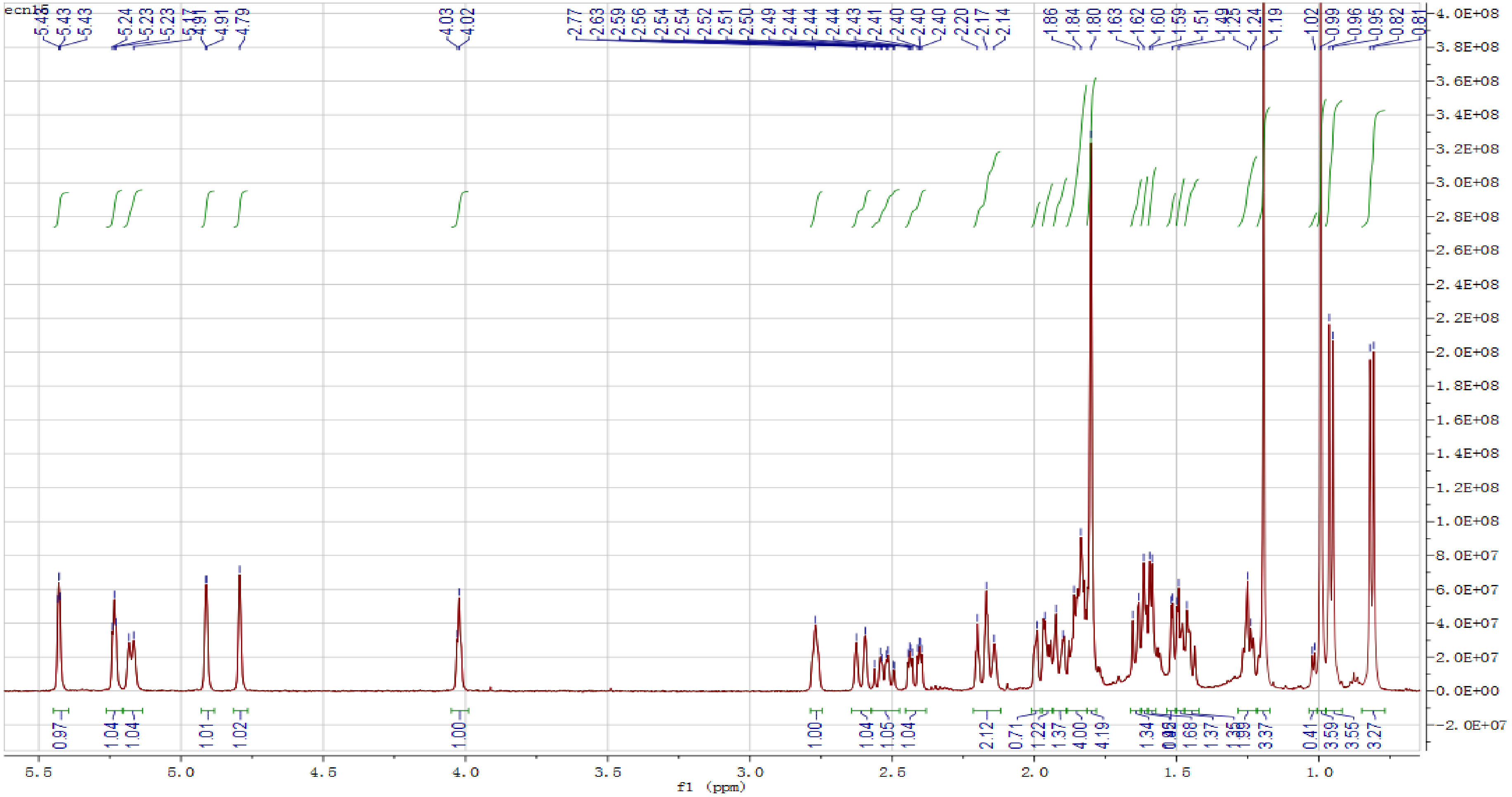

S15. ^{13}C NMR (DEPT 125 MHz) for wedelobatin B (**4**)

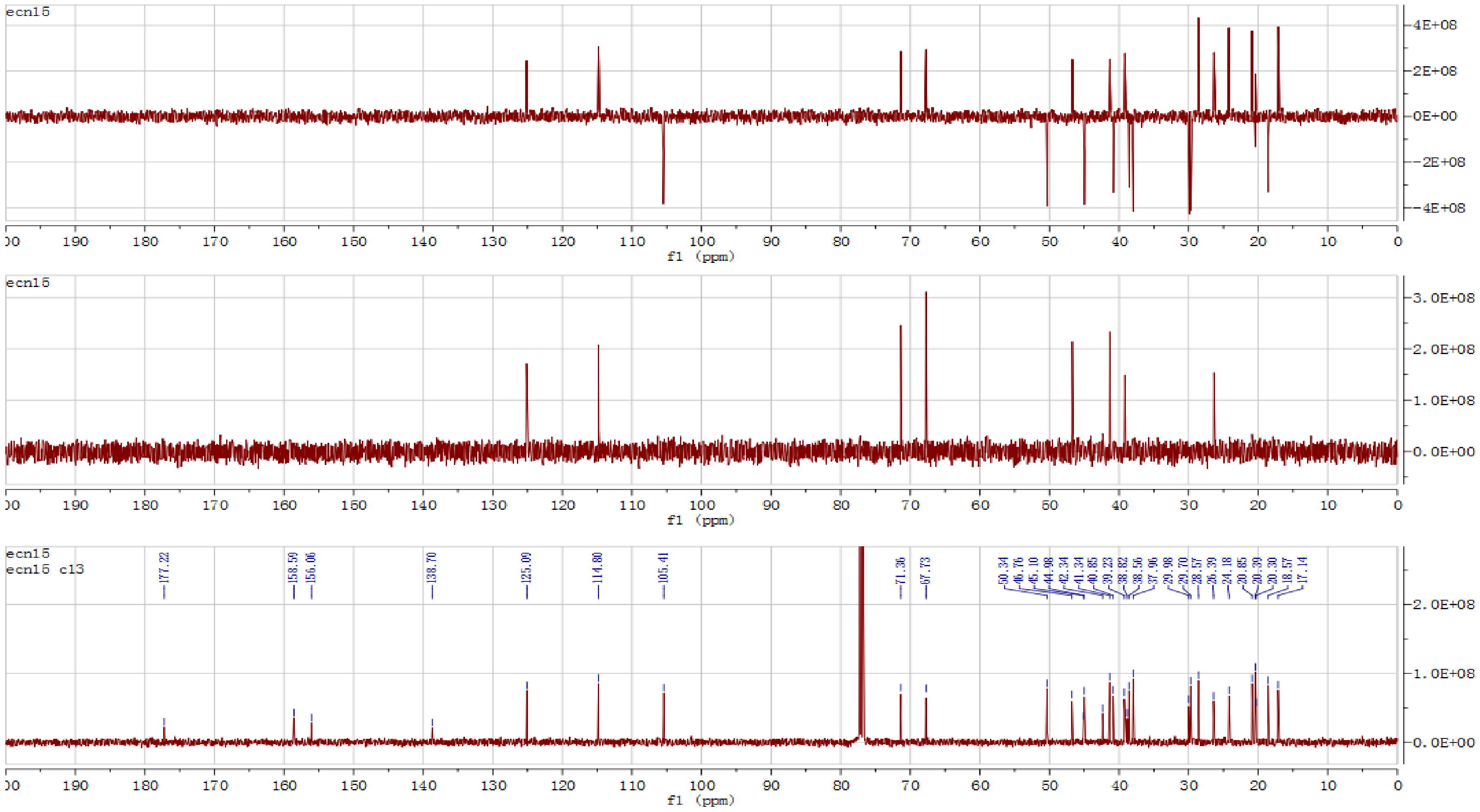

S16. HSQC (600 MHz) for wedelobatin B (**4**)

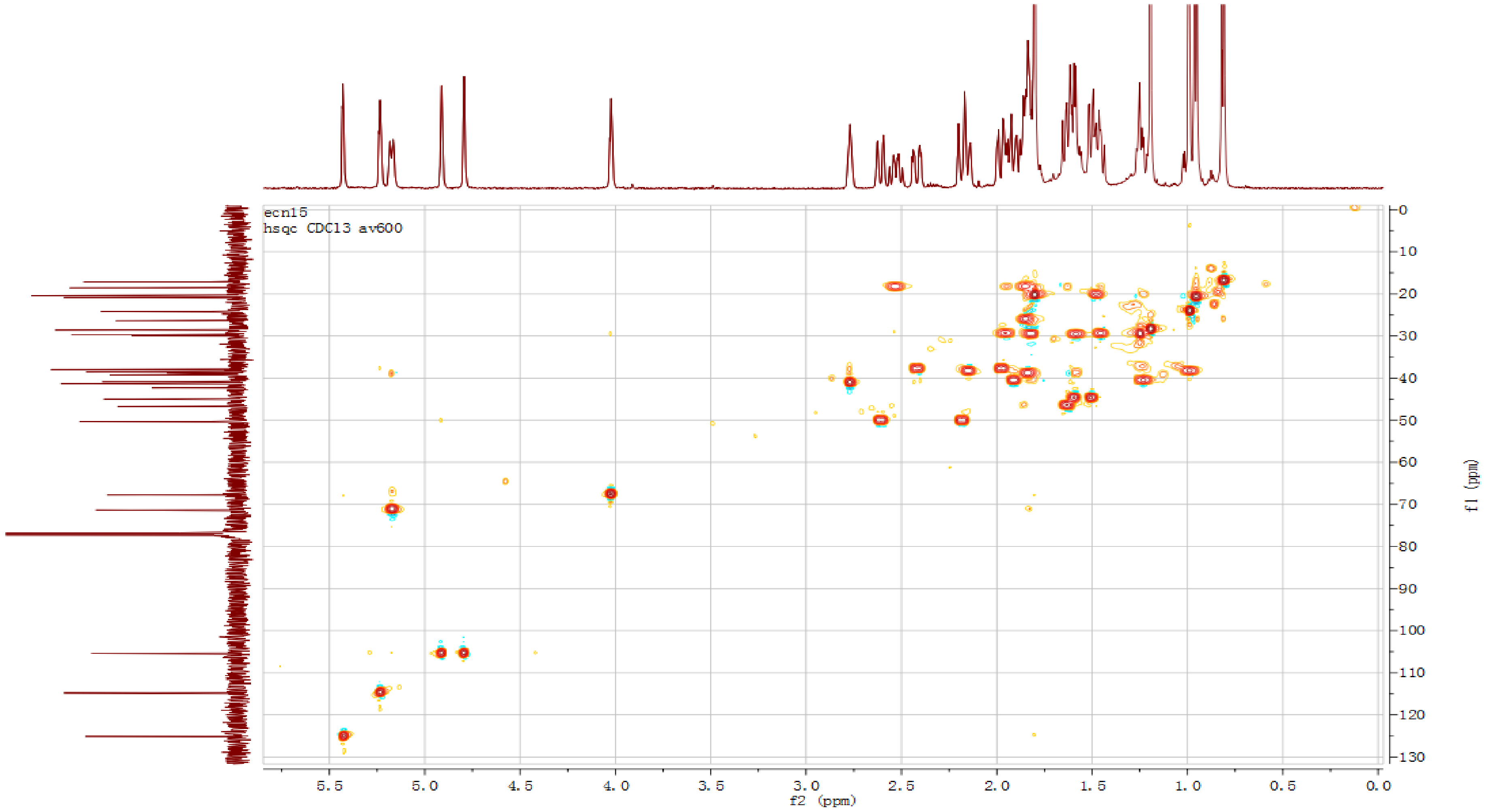

S17. HMBC (500 MHz) for wedelobatin B (**4**)

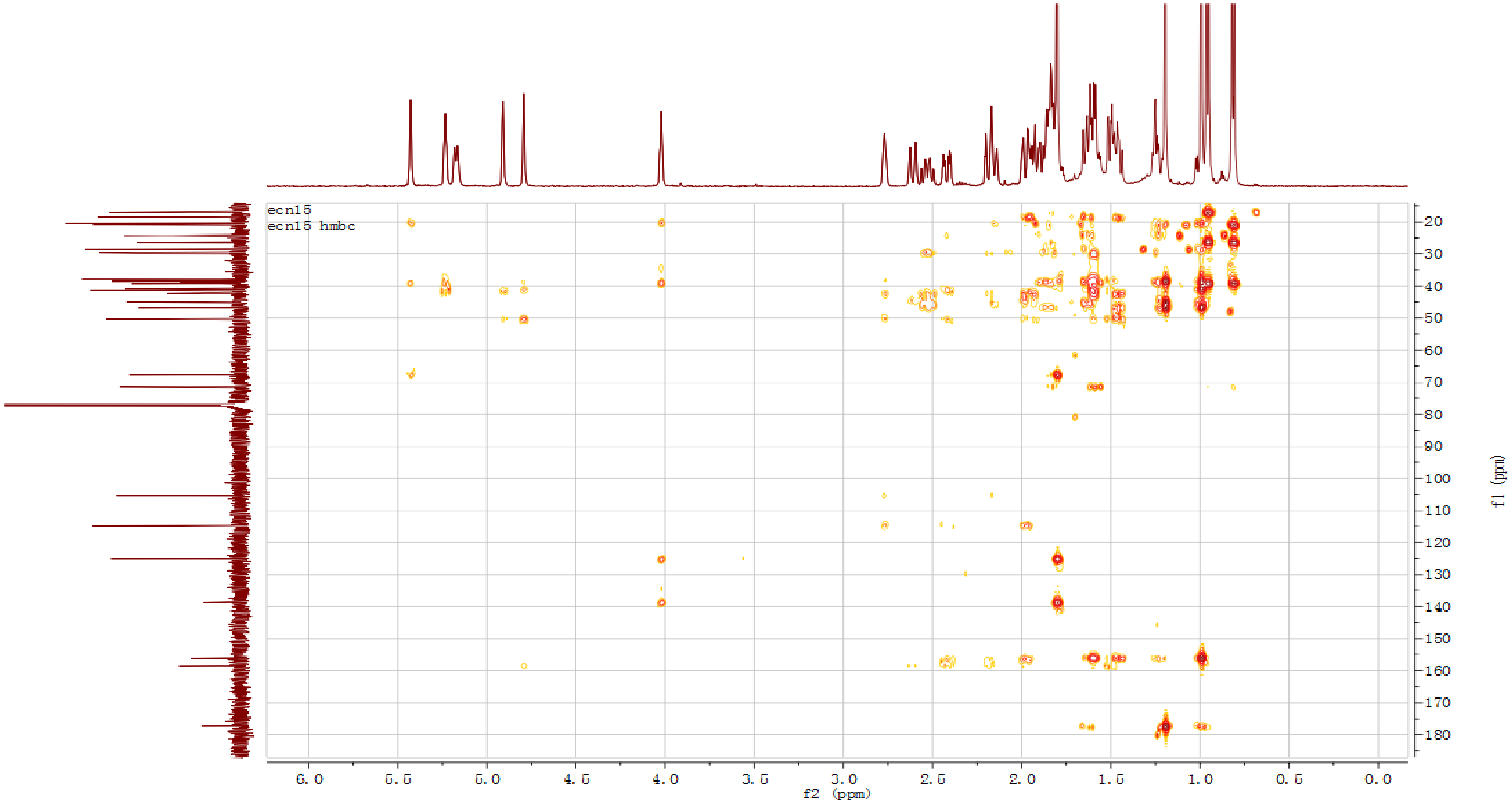

Supplement: Supplementary file 1 — Supplementary material, approximately 2.16 MB. [file 13659_2013_29_MOESM1_ESM.pdf]
